# The Transition from Primary siRNAs to Amplified Secondary siRNAs That Regulate Chalcone Synthase During Development of *Glycine max* Seed Coats

**DOI:** 10.1371/journal.pone.0076954

**Published:** 2013-10-21

**Authors:** Young B. Cho, Sarah I. Jones, Lila Vodkin

**Affiliations:** Department of Crop Sciences, University of Illinois, Urbana, Illinois, United States of America; Oregon State University, United States of America

## Abstract

The *I* locus is a 27-kb inverted repeat cluster of chalcone synthase genes *CHS1-3-4* that mediates siRNA down-regulation of *CHS7* and *CHS8* target mRNAs during seed development leading to yellow seed coats lacking anthocyanin pigments. Here, we report small RNA sequencing of ten stages of seed development from a few days post fertilization through maturity, revealing the amplification from primary to secondary short interfering RNAs (siRNAs) occurring during development. The young seed populations had a higher proportion of siRNAs representing the *CHS1-3-4* gene family members, consistent with this region as the origin of the primary siRNAs. More intriguingly, the very young seed had a higher proportion of 22-nt *CHS* siRNAs than did the mid-maturation seed. We infer that the primary *CHS* siRNAs increase during development to levels sufficient to trigger amplification of secondary *CHS* siRNAs from the *CHS7*/*8* target mRNAs, enabling the total levels of 21-nt *CHS* siRNAs to rise dramatically. Further, we demonstrate that the soybean system exhibits tissue-specific *CHS* siRNA production because primary *CHS* siRNA levels are not sufficient to trigger secondary amplification in tissues other than the seed coat.

## Introduction

Unique features of the gene silencing *I* (inhibitor) locus in soybean that paralleled co-suppression phenomena have been described [1] and the locus has been shown to be an example of naturally occurring RNA interference (RNAi) [2], [3]. The *I* locus produces tissue-specific siRNAs from an unusual arrangement of chalcone synthase (*CHS*) genes and these *CHS* siRNAs subsequently target *in trans CHS* mRNAs from genes located on different chromosomes [4].

The *I* locus was first identified as a region of duplicated and inverted *CHS* genes by DNA blotting and PCR mapping of 15 pairs of isogenic lines which showed that mutations of a dominant allele (*I* or *i^i^*) to the recessive allele (*i*) delete promoter sequences, paradoxically resulting in increased total *CHS* transcript levels and thereby pigmented (black) seed coats [1]. The inhibitory effects of this naturally occurring mutation appeared to parallel the co-suppressive phenotypes found in transgenic *Petunia* lines carrying multiple inserted copies of the *CHS* genes [5], [6]. Sequencing bacterial artificial chromosomes (BACs) from the Williams soybean variety containing the dominant *i^i^* allele revealed that five (*CHS1, CHS3, CHS4, CHS5,* and *CHS9*) of the nine *CHS* gene family members are located in a 230-kb region [7], [8]. *CHS1, CHS3*, and *CHS4* are located in two 10.91-kb perfect and inverted repeat clusters separated by 5.87 kb of intervening sequence.

The relative expression profiles of *CHS* gene family members were examined by quantitative RT-PCR in the seed coats of two near-isogenic pairs that result from independently occurring mutations of the dominant *I* allele to the recessive *i* allele or of the dominant *i^i^* allele to the recessive *i* allele [3]. Decreased expression of the *CHS7/8* genes results in the lack of pigmentation in the yellow seed coats (*I* and *i^i^*). Both small RNA blots and high throughput small RNA sequencing from three genotypes (*I, i^i^* and *i* alleles) of soybeans revealed that *CHS* siRNAs accumulated only in the yellow seed coats containing either dominant *I* or *i^i^* alleles and not in the pigmented seed coats with homozygous recessive *i* genotypes [4]. Interestingly, the *CHS* siRNAs were generated in a tissue-specific manner. *CHS* siRNAs did not accumulate in the cotyledons of the genotype with dominant *I* or *i^i^* alleles and yellow seed coats [4], thus allowing isoflavones to be produced in the cotyledons [9] since *CHS7/8* mRNAs are not down-regulated in the cotyledons.

The generation pathway of *CHS* siRNAs involved in silencing the *I* locus was proposed [4]. A putative double stranded (ds) RNA generated from within a 27-kb inverted *CHS* region comprised of two clusters of the *CHS1-3-4* and *CHS4-3-1* genes is cleaved into primary siRNAs representing both strands that are amplified by RNA-dependent RNA polymerase (RdRP) to generate secondary *CHS* siRNAs from the target *CHS7/8* mRNAs, which are capable of down-regulating all members of the *CHS* gene family. On the other hand, *CHS7/8* mRNAs are highly expressed in the pigmented seed coats in which *CHS* siRNA production has been abolished by deletions in the *CHS* cluster regions in the mutant *i* allele.

Small RNA sequencing of co-suppressed, non-pigmented transgenic petunia flowers with introduced *CHS* genes has shown that *CHS* siRNAs are the causative factor of silencing the pigment pathway in flowers [10]. There are many commonalities between the naturally occurring soybean seed coat and the transgenic petunia systems [11]. These similarities include *CHS* siRNAs that are predominantly 21 nt in size and match both sense and antisense strands primarily of exon 2 of the conserved target *CHS* genes, likely through amplification of the original signal through RdRP.

Here, we sequenced small RNA populations from a developmental series representing ten stages of seed development from a few days post fertilization through seed maturity. Due to the sequence polymorphisms between the *CHS* siRNA origin and target genes, we were able to reveal the amplification from primary to secondary *CHS* siRNAs that occurred early in development. The primary *CHS* siRNAs were lower in abundance but composed of a higher proportion of 22-nt small RNAs, whereas the more abundant secondary *CHS* siRNAs were primarily 21 nt in size. A developmental amplification from primary to secondary siRNAs has not previously been tracked in either naturally occurring or transgenic plant systems. We also demonstrate that *CHS* siRNA production is specific to the seed coats as other tissues including cotyledons, roots, leaves, and stems do not produce sufficient primary siRNAs for amplification to secondary siRNAs.

## Results

### Differential Expression of *CHS* siRNAs during Seed Development

**Figure 1:**
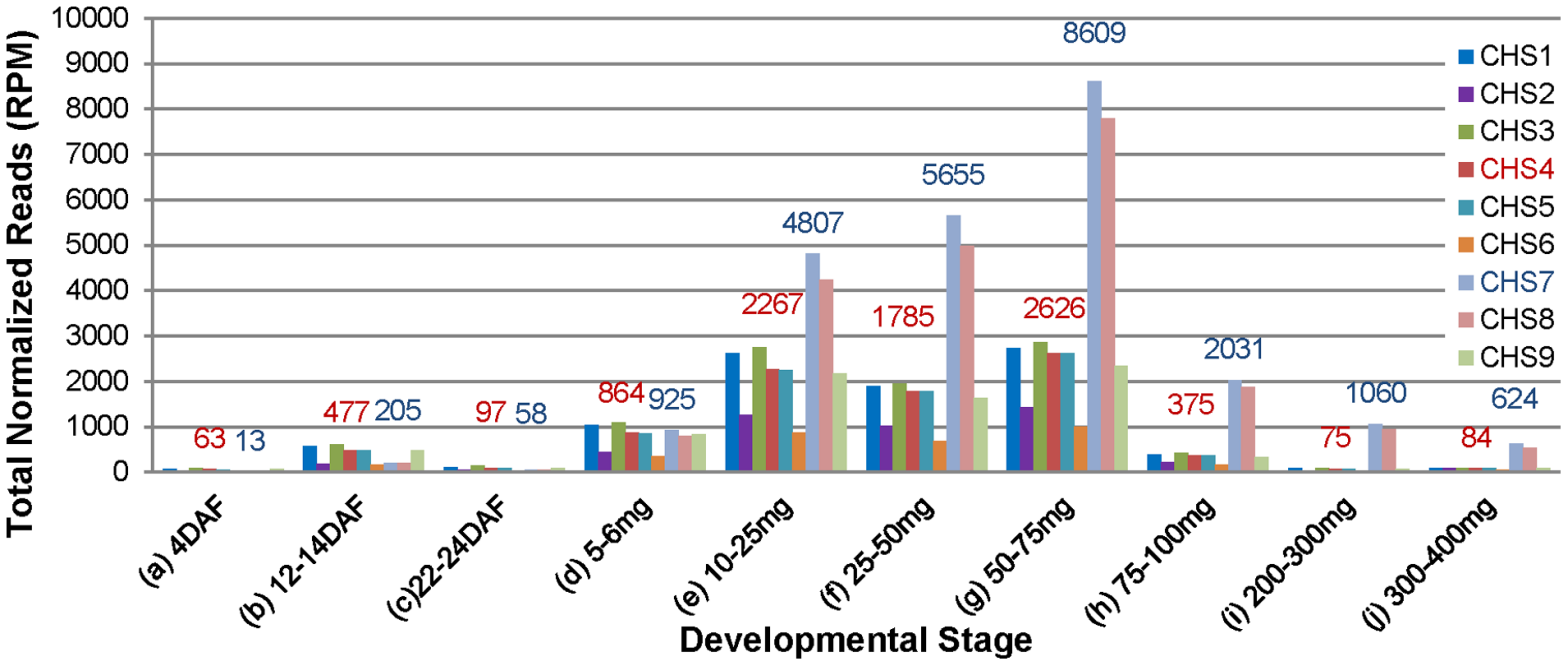
Total Counts of *CHS* siRNAs for Each *CHS* Gene Reveal *CHS7/8* siRNAs are Dramatically Increased During Seed Coat Development. *CHS* siRNAs from small RNA libraries of ten developmental stages of the cultivar Williams *i^i^* were filtered to identify those with 100% identity to individual *CHS* genes as indicated by the color chart. RPM, reads per million. Numbers above the bar indicate the total counts of *CHS4* (red) and *CHS7* (blue) siRNAs. Developmental stages are whole seed from (a) 4 DAF (Days After Flowering); (b) 12–14 DAF; and (c) 22–24 DAF; seed coats dissected from immature green seed of fresh weight (d) 5–6 mg; (e) 10–25 mg; (f) 25–50 mg; (g) 50–75 mg; (h) 75–100 mg; (i) 200–300 mg; and seed coats from (j) 300–400 mg yellow, desiccating seed.

Although *CHS1, 2, 3, 4, 5, 6* and *9* share 93% to 98% pairwise identity by nucleotide sequence, they are only 82% similar to *CHS7* and *CHS8*. The dispersed nucleotide polymorphisms between these two groups of *CHS* genes present the opportunity to distinguish siRNAs representing the *CHS7/8* mRNAs that are down-regulated *in trans* by *CHS1/3/4* primary siRNAs originating from the *I* locus, which is defined by inverted repeats of *CHS1, CHS3* and *CHS4* genes (referred to as the *CHS1-3-4* clusters). To determine the pattern of *CHS* siRNAs during the entire span of seed development, we constructed small RNA libraries from ten stages of seed development of the cultivar Williams (*i^i^*) as shown in Figure 1. In the early developmental periods from 4 DAF (days after flowering) through 22–24 DAF, whole seeds were used as the maternal seed coat is proportionally a larger part of the whole seed, which is too small for hand dissection. Beginning at the 5–6 mg weight range, the developmental staging is more accurate using a combination of seed fresh weight and color changes. At that time the seed are large enough to dissect the seed coat free of the cotyledons and embryonic axis. Table 1 lists details on the small RNA sequencing, ranging generally from 10 to 30 million reads from each of the ten developmental stages. Two biological repeats were made for eight of the ten developmental stages.

**Table 1:** Summary Information on Eighteen Sequenced Small RNA Libraries from a Developmental Series of Williams (PI548631) Seed and Seed Coats Including Developmental Stage and Number of Reads.

Tissue	Developmental stage	Replicate	Instrument model	# of Reads
**Whole Seed**	4 days after flowering	BR1	Illumina HiSeq 2000	27.0 M
**Whole Seed**	4 days after flowering	BR2	Illumina HiSeq 2000	12.1 M
**Whole Seed**	12–14 days after flowering	BR1	Illumina HiSeq 2000	35.2 M
**Whole Seed**	12–14 days after flowering	BR2	Illumina HiSeq 2000	13.1 M
**Whole Seed**	22–24 days after flowering	BR1	Illumina HiSeq 2000	30.8 M
**Whole Seed**	22–24 days after flowering	BR2	Illumina HiSeq 2000	10.3 M
**Seed Coat**	5–6 mg fresh weight	BR1	Illumina HiSeq 2000	17.8 M
**Seed Coat**	5–6 mg fresh weight	BR2	Illumina HiSeq 2000	25.3 M
**Seed Coat**	10–25 mg fresh weight	NA	Illumina HiSeq 2000	12.0 M
**Seed Coat**	25–50 mg fresh weight	NA	Illumina HiSeq 2000	12.0 M
**Seed Coat**	50–75 mg fresh weight	BR1	Illumina Genetic Analyzer	*2.9 M
**Seed Coat**	50–75 mg fresh weight	BR2	Illumina HiSeq 2000	11.5 M
**Seed Coat**	75–100 mg fresh weight	BR1	Illumina Genome Analyzer II	30.1 M
**Seed Coat**	75–100 mg fresh weight	BR2	Illumina HiSeq 2000	10.5 M
**Seed Coat**	200–300 mg fresh weight	BR1	Illumina HiSeq 2000	31.3 M
**Seed Coat**	200–300 mg fresh weight	BR2	Illumina HiSeq 2000	14.8 M
**Seed Coat**	300–400 mg fresh weight	BR1	Illumina Genome Analyzer II	34.0 M
**Seed Coat**	300–400 mg fresh weight	BR2	Illumina HiSeq 2000	17.1 M

The PI number is an accession number by which information on the cultivar is searchable in the USDA GRIN (Germplasm Resources Information Network). Williams is homozygous dominant *i^i^* genotype with yellow seed coats.

* This library was previously reported in [4]. BR1 and BR2 are biological repeats; NA, not applicable.

Figure 1 shows the normalized total counts in reads per million (RPM) of *CHS* siRNAs that map with 100% identity to each of the nine *CHS* genes during seed development. The normalized *CHS* siRNA counts were initially low in the 4 DAF seed at less than 100 RPM and also in the 12–14 DAF and 22–24 DAF seed. Then, they increased to about 900 RPM in seed coats from immature seed of 5–6 mg fresh weight, after which they increased dramatically, reaching the highest levels of 8600 for *CHS7* in seed coats from 50–75 mg immature seed. After this peak, the *CHS* siRNAs decreased as the seed increased in size and began desiccation. An analysis of *CHS* siRNAs from a biological repeat (Figure S1) of eight of the ten stages shows the same pattern as Figure 1 with maximal *CHS* siRNAs found in the 50–75 mg stage.

Strikingly, the expression pattern of Figure 1 and Figure S1 enables us to distinguish the primary siRNAs originating from the *CHS1-3-4* clusters of the *I* locus from those representing secondary siRNAs matching target mRNAs encoded by *CHS7* and *CHS8*. The secondary *CHS7/8* siRNAs showed an increase in the 5–6 mg seed coats and peaked at the 50–75 mg stages. The predominance of secondary *CHS7/8* siRNAs remained during the rest of development in seed coats dissected from the immature green seed of 75–100 mg and 200–300 mg, as well as from 300–400 mg yellow seed coats that were undergoing desiccation. These data demonstrate that the amplification from primary to secondary *CHS* siRNAs occurs during seed coat development.

### Primary siRNAs are Predominant before Accumulation of 21-nt Secondary siRNAs

**Figure 2:**
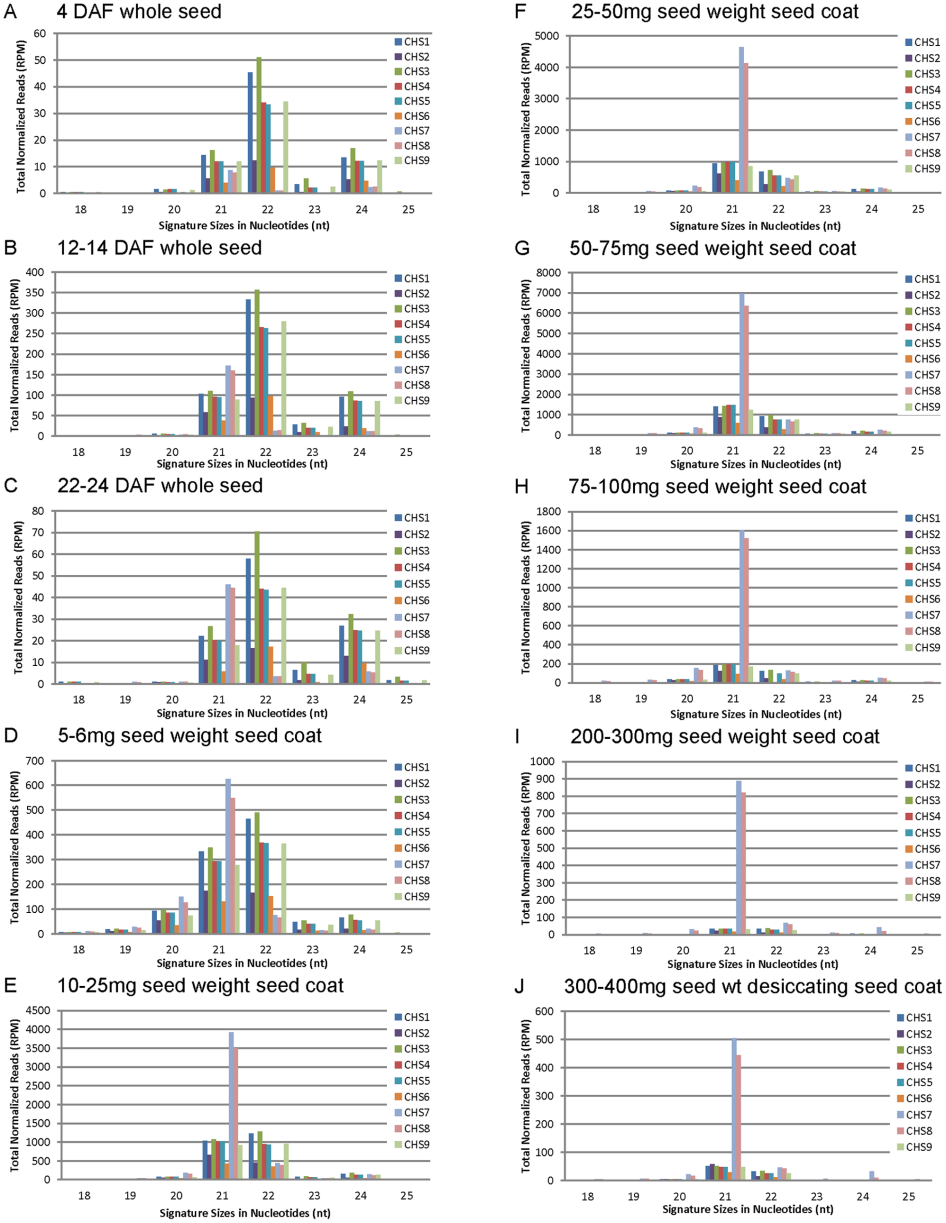
Size Distributions of *CHS* siRNAs Reveal the Amplification of 21-nt Secondary siRNAs During Seed Coat Development. *CHS* siRNAs from small RNA libraries of the same ten developmental stages A-J as described in Figure 1 were analyzed for their size distributions between 18 and 25 nt. The normalized total counts in reads per million (RPM) are shown for each *CHS* gene according to the color chart.

To investigate characteristics of secondary *CHS* siRNAs which are amplified during development, an analysis of the size distribution of *CHS* siRNAs was conducted. Figure 2 clearly shows that *CHS7/8* siRNAs, which increased dramatically during development, were 21 nt in size. To the contrary, the *CHS1/3/4* siRNAs were predominantly 22 nt in size up to the 5–6 mg stage (Figure 2). These data suggest that secondary *CHS* siRNAs were predominantly 21 nt in size and that the primary siRNAs consist of a slightly higher proportion of 22-nt siRNAs. However, in the early developmental stages, 21-nt primary *CHS* siRNAs are also actively generated along with the 22-nt size class. Similar results were found for the biological repeat (Figure S2).

**Figure 3:**
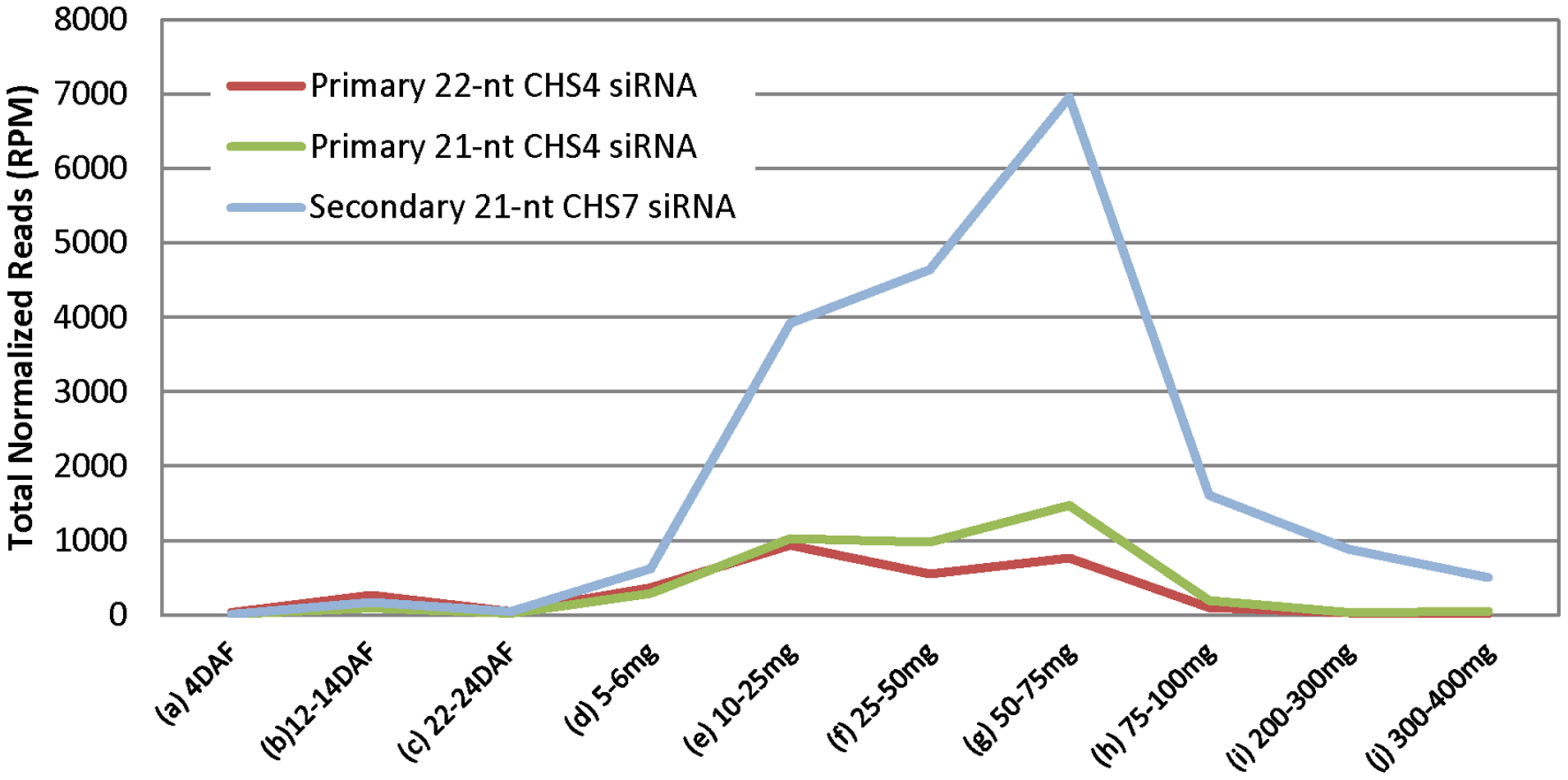
Normalized Total Counts of *CHS* siRNAs that Match Either *CHS4* or *CHS7* with 100% Identity During Ten Stages of Seed Coat Development. The 21-nt secondary *CHS7* siRNAs are amplified to high levels relative to the *CHS4* 21-nt or 22-nt siRNAs. Developmental stages are same as described in Figures 1 and 2.

A comparison of total counts of 21-nt and 22-nt primary *CHS4* siRNAs and 21-nt secondary *CHS7* siRNAs that are unique to either *CHS4* or *CHS7* shows that 21-nt secondary siRNAs were highly amplified during seed coat development compared to those of primary *CHS* siRNAs (Figure 3 and Figure S3). RdRPs are involved in RNA amplification of primary siRNAs and generate secondary siRNAs [12]–[14]. In the soybean seed coat, we demonstrate that this amplification started at an early developmental stage around 5–6 mg (Figures 2 and 3) likely at the stage when *CHS7*/*8* mRNAs begin to be expressed in the seed coats at higher levels. The *CHS7/8* mRNAs are then susceptible to degradation by primary siRNAs, and amplification is guided by the primary *CHS1/3/4* siRNAs that are produced initially from the triggering clusters of the *CHS1-3-4* genes of the dominant *i^i^* allele.

### High Throughput Small RNA Sequencing Verifies Tissue Specificity of *CHS* siRNAs

Using mRNA blots and small RNA blots, we have previously shown that silencing of *CHS* gene family members occurs only in the seed coats and not in other organs [3], [4]. Using sequencing, small RNAs were detected in significant numbers only in the dissected seed coats and not the cotyledons of the developing seed [4] from the 50–75 mg weight range. Here, we investigated additional tissues such as leaf, root and germinating cotyledon with the power of deep sequencing to ascertain the tissue specificity of *CHS* siRNAs. As shown in Table 2, total numbers of small RNA reads were from three million to thirty million from each of the tissues from Williams cultivar or other cultivars with *i^i^* genotype, providing sufficient data to show the tissue specificity of *CHS* siRNAs and to examine even low levels of *CHS* siRNAs.

**Table 2:** Summary of Small RNA Libraries Constructed from Various Williams (PI548631) Tissues Including Developmental Stage and Number of Reads.

Tissue	Replicate	Developmental stages	Instrument model	Reads
**Seed Coat**	NA	5–6 mg fresh weight	Illumina HiSeq 2000	25.3 M
**Immature Cotyledon**	BR1	5–6 mg fresh weight	Illumina HiSeq 2000	31.5 M
**Immature Cotyledon**	BR2	5–6 mg fresh weight	Illumina HiSeq 2000	31.1 M
**Immature Cotyledon**	NA	50–75 mg fresh weight	Illumina Genetic Analyzer	*3.0 M
**Immature Cotyledon**	NA	100–300 mg fresh weight	Illumina HiSeq 2000	31.9 M
**Germinated Cotyledon**	NA	10 Day Seedling	Illumina Genome Analyzer II	16.4 M
**Leaf**	NA	10 Day Seedling	Illumina HiSeq 2000	15.2 M
**Root**	NA	10 Day Seedling	Illumina Genome Analyzer II	17.3 M
**Shoot Tip**	NA	10 Day Seedling	Illumina HiSeq 2000	15.7 M
**Stem**	NA	10 Day Seedling	Illumina HiSeq 2000	22.3 M

The PI number is an accession number by which information on the cultivar is searchable in the USDA GRIN (Germplasm Resources Information Network). Williams is homozygous dominant *i^i^* genotype with yellow seed coats.

* This library was previously reported in [4].

**Figure 4:**
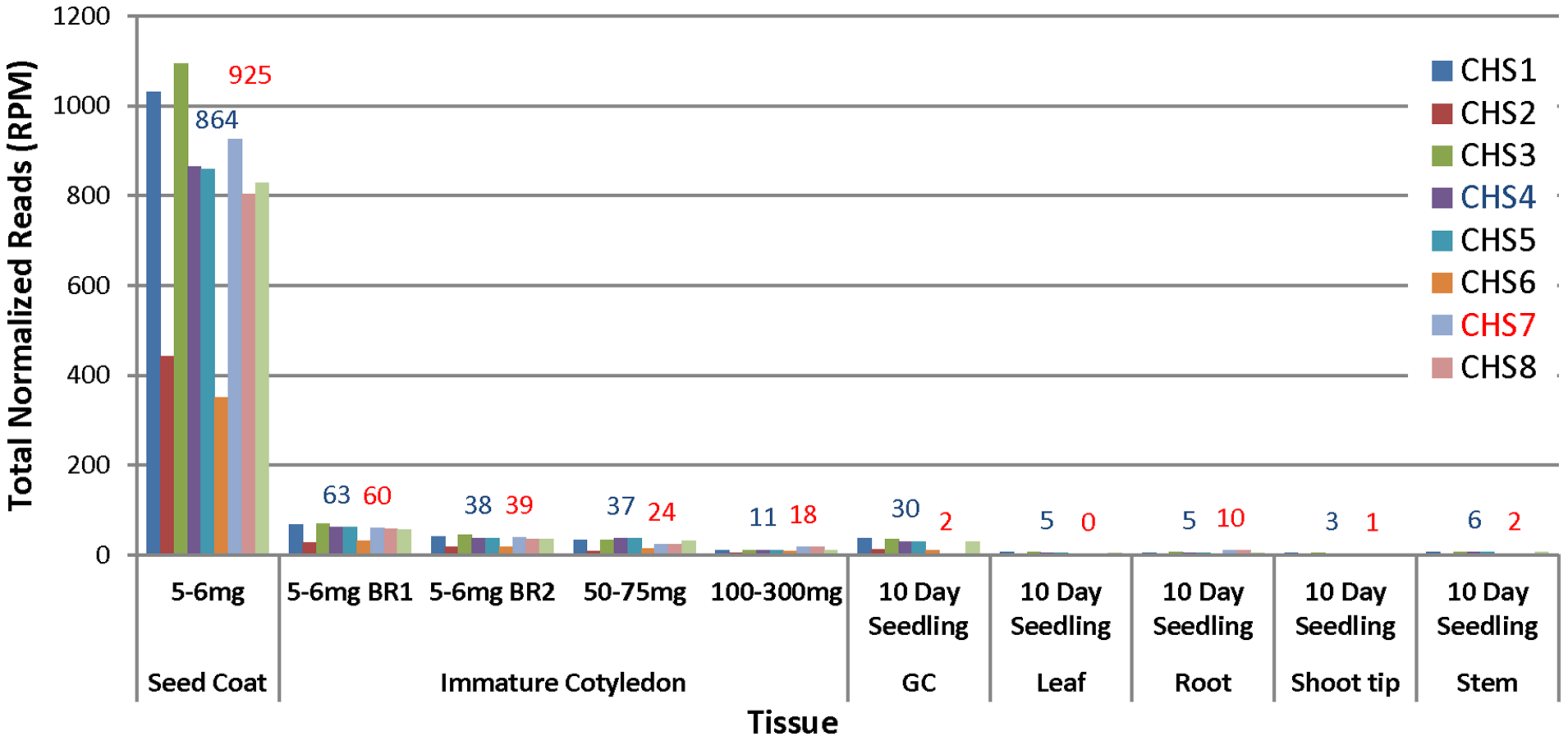
The Total Counts of *CHS* siRNA Family Members in Seven Different Soybean Tissues with *i^i^* Genotype Show Very Low Levels Except in the Yellow Seed Coat. Numbers above the bar indicate the total normalized counts of *CHS4* (red) and *CHS7* (blue) siRNAs. BR1 and BR2 are biological repeats which were grown at different times. RPM, reads per million. GC stands for germinated cotyledon.

Figure 4 demonstrates the tissue specificity clearly as *CHS* siRNAs were limited to the seed coats in the Williams *i^i^* cultivar. The normalized total counts of *CHS7* siRNAs in seed coats from 5–6 mg is 925 (RPM). In contrast, the expression levels of *CHS7* siRNAs were less than 60 counts in four other tissues which is approximately a 15-fold difference (Figure 4). Most of those tissues (immature cotyledon, germinated cotyledon, unifoliate, root, shoot tip, and stem) have more *CHS4* siRNAs than *CHS7* siRNAs; however the counts of *CHS4* siRNAs are very low compared to those of seed coat tissues (Figure 4). These data show that both primary and secondary *CHS* siRNAs were not significantly produced in germinated cotyledon, immature cotyledon, leaf, root, shoot tip, and stem tissues.

Seed coat tissue is close to the cotyledon physically during seed development. It is interesting that *CHS* siRNAs were limited to the seed coat and were not in immature cotyledons of three different stages of development, 5–6 mg, 50–75 mg and 100–300 mg fresh seed weight. The immature cotyledons contained very few *CHS* siRNAs in these three developmental stages (Figure 4). Because of the small size of the seed prior to the 5–6 mg stage, we used whole seed through 24 DAF; thus, it is likely also that the low levels of *CHS* siRNAs from these young whole seed less than 24 DAF (Figure 1) originated solely from the maternal seed coat tissue and not from the very young developing cotyledons since the cotyledons at all succeeding stages did not accumulate *CHS* siRNAs at significant levels as shown in Figure 4.

## Discussion

### The Amplification from Endogenous Primary to Secondary *CHS* siRNAs was Revealed by Small RNA-Seq of a Broad Range of the Developing Seed Coats

**Figure 5:**
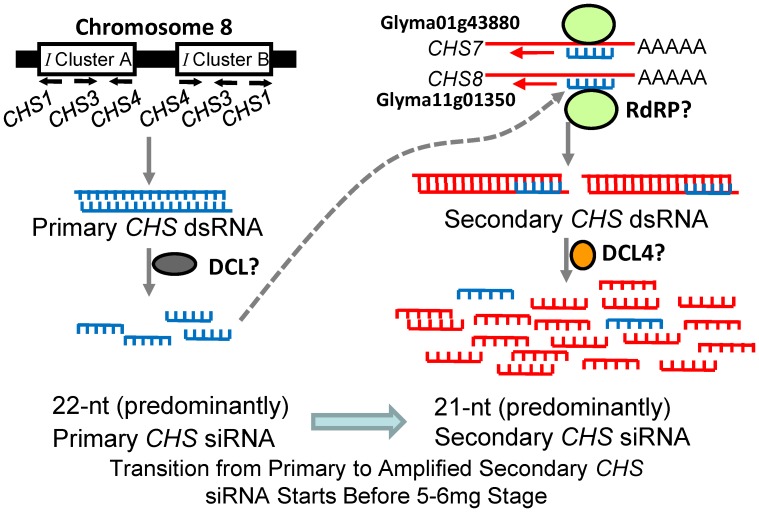
Biogenesis of *CHS* siRNAs Preventing Pigment Formation in Developing Soybean Seed Coats of the Williams Cultivar with *i^i^* Genotype. Partially adapted from [4]. The long inverted repeat of the *i^i^* allele of the *I* locus on Chromosome 8 contains cluster A (*CHS1-3-4*) and cluster B (*CHS4-3-1*) and forms the nascent *CHS* dsRNA although the exact size is unknown. The cleavage of *CHS* dsRNA by putative Dicer Like proteins (DCL) generates predominantly 22-nt primary *CHS* siRNAs at the earliest developmental stages, resulting in the degradation *in trans* of targeted transcripts from *CHS7* (Glyma01g43880) and *CHS8* (Glyma11g01350) located on Chromosomes 1 and 11, respectively. After cleavage at the mRNA sites targeted by the primary *CHS* siRNAs, a putative RdRP synthesizes dsRNA from the cleaved *CHS* mRNA. The 21-nt secondary *CHS* siRNAs likely generated by DCL4 homologs from this dsRNA can target additional *CHS* mRNAs, thus amplifying the silencing response as well as spreading it over a larger region of the target. The transition from predominantly 22-nt *CHS* siRNAs (shown in blue) representing the origin of the *CHS1-3-4* siRNAs to the 21-nt secondary *CHS* siRNAs (shown in red) representing the target *CHS7* and *CHS8* mRNAs occurs at the 5–6 mg seed stage as indicated.

We have previously presented evidence for a model of action for the naturally occurring dominant *I* and *i^i^* alleles in preventing pigment formation in soybean seed coats [4] based on knowledge of RNA interference mechanisms in other organisms [12], [13]. Figure 5 summarizes the model and illustrates our data from this report on the developmental transition from primary to secondary siRNAs. The long inverted repeat of the *I* locus on Chromosome 8 contains cluster A (*CHS1-3-4*) and cluster B (*CHS4-3-1*) and forms the nascent *CHS* dsRNA although the exact size is unknown. The cleavage of *CHS* dsRNA by Dicer Like proteins (DCL) generates the primary *CHS* siRNAs, resulting in the degradation *in trans* of targeted *CHS7* and *CHS8* transcripts that originate from Chromosomes 1 and 11, respectively. After cleavage at the mRNA sites targeted by the primary *CHS* siRNAs, an RdRP synthesizes dsRNA from the cleaved *CHS* mRNA. The secondary *CHS* siRNAs generated from this dsRNA could target additional *CHS* mRNAs, amplifying the silencing response as well as spreading it over a larger region of the target. The roles for putative DCL and RdRP like functions in soybean are based on extrapolation from other systems and not by analysis of mutants of these functions in soybean.

In this report, we show that the unique attributes of the naturally occurring soybean *I* locus clearly revealed the amplification of the *CHS* siRNA population from primary to secondary siRNAs during seed development based on the polymorphisms between the siRNAs representing the origin (*CHS1-3-4*) and target *CHS7* and *CHS8* genes. Table S1 shows that very few siRNAs match both the origin and target subgroups with 100% identity, verifying that the observed patterns (Figures 1, 2, 3 and Figures S1, S2, S3) are not due to multi-matching siRNA. The polymorphisms between the two sub-groups are widely dispersed throughout the sequence. For example, although *CHS4* and *CHS7* are 82% identical [4], there are no siRNAs which are longer than 20 nt matching both *CHS4* and *CHS7* at 100% identity as shown in the alignment of Figure S4. Allowing up to two-base mismatches between the origin and target increases the number of siRNA sequences significantly (Table S1). Thus, the targeting of *CHS7* and *CHS8* by the *CHS1-3-4* primary siRNAs is likely primarily through mismatching rather than perfectly matched siRNAs.

A developmental transition from primary to secondary siRNAs has not previously been elucidated in either naturally occurring or transgenic plant systems. In *C. elegans*, deep sequencing distinguished primary and secondary siRNAs using an exogenous RNAi construct containing mismatches from the wild type *C. elegans* target sequence positioned at 25 base intervals [15]. Here, by examining the entire seed developmental period including the very early stages from a few days post fertilization that are labor intensive to obtain in sufficient quantity for sequencing, we demonstrated the transition from a small population of primary *CHS* siRNAs to the accumulation of high levels of 21-nt secondary siRNAs (Figures 1, 2, 3 and Figures S1, S2, S3). The siRNA populations in the young seed had a higher proportion representing the *CHS1-3-4* gene family members, consistent with this region as the origin of the primary siRNAs. Seed at 4 DAF contained a basal level of less than 100 RPM that aligned to any *CHS* gene. The levels of *CHS* siRNAs originating from the *CHS1-3-4* clusters increased thereafter to roughly 400–900 RPM very early in seed development from 12–24 DAF to 5–6 mg. By the 5–6 mg stage, the seed coats contained roughly even proportions of siRNAs representing *CHS1-3-4* compared to *CHS7* and *CHS8* but after that period, the *CHS7/8* siRNAs increased dramatically, reaching peak levels of 8,000 to 10,000 RPMs in the biological repeats of the 50–75 mg seed coats.

Intriguingly, the very young seed had a higher proportion of 22-nt *CHS* siRNAs than did seed from the 5–6 mg stage and older. Recent studies in *Arabidopsis thaliana* [16]–[18] demonstrated that 22-nt miRNAs (microRNAs) are critical for biogenesis of secondary 21-nt siRNAs as they drive amplification of secondary siRNAs via RdRP to generate ta-siRNAs (transacting siRNAs). Our data support the conclusions that the primary siRNA population has a higher proportion of 22-nt siRNAs, and that the secondary population amplified from the *CHS7/8* substrates by RdRP are predominantly 21 nt in size. However, another study recently claimed that the miRNA-duplex structure is critical to the production of secondary ta-siRNAs rather than the length of miRNA [19]. Whether or not the 22-nt siRNAs are the only trigger to generate the 21-nt secondary siRNAs is unknown, since 21-nt primary *CHS* siRNAs were also present in the very young seed. The predominance of the 21-nt secondary siRNAs that represent *CHS7/8* was maintained as the total levels of *CHS* siRNAs declined during the later stages of maturity.

### Biogenesis of *CHS* siRNAs is Regulated by the Developmental Program in Soybean Seed

*CHS* siRNAs were found to be most abundant in the 50–75 mg stage among ten developmental stages (Figures 1 and 2). Previously, it was shown with RNA blots that *CHS* mRNAs are prevalent at 10–25 mg, 25–50 mg and 50–75 mg weight ranges of the seed coats of mid-maturation embryos with pigmented genotypes [3]. Also, using TaqMan real time RT-PCR, it was shown that *CHS7* and *CHS8* mRNAs are highest at the 50–75 mg stage of Williams 55 pigmented seed coats with the homozygous recessive *i* genotype. In this regard, the profile of the *CHS* siRNAs resembled many other genes expressed during seed coat development, including many in the isoflavone and anthocyanin pathways [20]. The most abundant levels of *CHS* siRNAs in the *i^i^* genotype with yellow seed coats generally coincided with the developmental time of appearance of the *CHS7* and *CHS8* target mRNAs in the recessive *i* genotype that has pigmented seed coats. Thus, in contrast with the down-regulation of the pathway by *CHS* siRNA-targeted destruction of *CHS* mRNAs in the yellow seed coats, the *CHS* transcripts continued to produce *CHS* isoforms after the 50 mg stage with the resulting accumulation of large amounts of anthocyanins leading to the pigmented seed coat.

By examining the entire seed developmental period including the small seed at 4 DAF through 24 DAF, we found that the *CHS* primary siRNAs representing the *CHS1-3-4* origin clusters began to increase before the secondary siRNAs representing the *CHS7/8* target mRNAs (summarized in Figure 5). These levels were higher than the basal level for *CHS* siRNAs found in non-seed tissues (Figure 4). From these results, we deduce that there was active biogenesis of the nascent *CHS* dsRNA that began to occur early in development between 12–24 DAF. It is not yet determined how early the *CHS7* and *CHS8* mRNAs begin to be expressed in the non-silencing, pigmented mutant line Williams (*i*, black). Although RNA-Seq data is available from the Williams (*i^i^*, yellow) variety at these early stages [21], it is not yet available from the pigmented mutant line Williams (*i*, black).

### Primary *CHS* siRNA Levels are Not Sufficient to Trigger Secondary Amplification in Tissues Other than the Seed Coat

Several hypotheses have been put forward to explain the tissue-specific accumulation of *CHS* siRNAs [3], [4]. (1) One mechanism could involve the action of a cell or tissue-specific transcription factor or DNA binding protein that initiates sufficient production of the dsRNA progenitor molecules only in the seed coats and not in other tissues of varieties with the dominant *I* and *i^i^* alleles. Alternatively, (2) the dsRNA could be formed in other tissues but not cleaved properly by a DCL protein to yield enough primary siRNA to trigger secondary siRNAs, or (3) the primary *CHS* siRNAs might not be amplified to high levels of secondary siRNAs due to lack of an RdRP or other core components to generate secondary siRNAs in other tissues. Here, our results with high throughput sequencing of large populations of small RNAs from eight non-seed tissues revealed only very low basal levels of *CHS* siRNAs, generally in the range of 60 RPM. We propose that the level of the *CHS* primary siRNAs is too low to trigger the secondary amplification in these tissues, unlike the situation in the non-pigmented seed coats where our data clearly show a rise in the levels of the primary *CHS* siRNAs to nearly 1000 RPM before the secondary siRNAs overtake them in abundance.

Using RNAse protection experiments, a recent study has detected *CHS* dsRNA not only in the seed coat but also in the cotyledon and leaf tissues of lines with a dominant *I* allele [22]. Although those results were not quantitative, they suggested that the biogenesis of *CHS* siRNA could be regulated in a tissue-specific manner after dsRNAs are generated by the failure to amplify secondary siRNAs because of a lack of a critical component in the amplification step, such as an RdRP activity. However, this would not explain why *CHS* dsRNAs that are formed in non-seed coat tissues would fail to be processed and amplified when many other miRNAs and siRNAs are fully processed and amplified in other tissues such as cotyledons, leaves, and roots of soybean with the *I* and *i^i^* alleles [4], [23], [24] including numerous phased siRNAs (phasiRNAs) resulting from 22-nt miRNAs in soybean roots that target the *NB-LRR* defense gene families [25]. As shown in Figures 1, 2, 3, 4, the amplification of the secondary siRNAs starts in the 5–6 mg seed coats. Recently, RNA-Seq data of seed coat and cotyledons from the 5–6 mg stage has been reported [21]. The RNA-Seq data showed that the expression levels of Glyma models with annotations such as RdRP, DCL, and AGO (argonaute like protein), are very low and have no significant differences between seed coat and cotyledon (data not shown). For these reasons, we currently prefer hypothesis (1) that the level of the *CHS* siRNAs follows an increase in the nascent dsRNA to levels that are higher in the seed coats than in other tissues.

Whether the limiting factor proves to be the levels of the dsRNA or primary siRNAs in tissues other than the seed coat, it is clear that secondary amplification from the target *CHS* mRNAs does not occur in other tissues as seen by the lack of high levels of 21-nt *CHS* siRNAs that align to any of the *CHS* genes (Figure 4). The absence of secondary siRNAs is not from the lack of *CHS* mRNA substrates as can be seen from a number of studies that report reasonable levels of *CHS* mRNAs in cotyledons, roots, leaves, and other tissues using RNA blots [3]. In addition, using RT-PCR, most of the *CHS* genes including *CHS7* and *CHS8* were shown to be induced to high levels in pathogen-challenged leaf tissues [26] indicating that sufficient *CHS* mRNA substrates for secondary amplification from even a low level of primary *CHS* siRNAs in the leaves would be possible. It is clear that *CHS* is down-regulated by the *CHS* siRNAs only in the seed coats, though sufficient levels of *CHS* mRNAs exist in other tissues to serve as substrates for amplification of secondary siRNAs to high levels.

In summary, because of the unique properties of the soybean silencing system, we were able to reveal that the onset of production of the primary *CHS* siRNAs in the very early seed stages contained a higher proportion of 22-nt siRNAs originating from the *CHS1-3-4* clusters of the *i^i^* allele. Then, the transition during development to a very large population of 21-nt secondary siRNAs representing amplification from the target *CHS7* and *CHS8* genes was apparent. Our results demonstrate regulation of the *CHS* inverted repeat genomic region is likely to rely on factors that occur during the very early seed developmental program and may reflect such changes. Further study of this system should provide more insight into the mechanisms of tissue specificity regulated by small RNAs and more broadly the blueprint of genes involved in development using the seed coat as a model.

## Methods

### Plant Materials, Tissue Collection, and Small RNA Isolation

The soybean line used in this study is inbred and homozygous for the indicated loci and is available from the USDA germplasm collection through GRIN (Germplasm Resources Information Network). The variety Williams (PI 548631), maturity group III, has genotype *i^i^* with a black hilum on an otherwise yellow seed coat. The genome of the closely related Williams 82 isoline was recently sequenced [27] and is used as a reference genome standard in soybean. The PI number is an accession number by which information on the cultivar is searchable in the GRIN database.

Soybean plants were grown in the greenhouse and immature seeds were harvested over the course of several weeks. The three earliest stages were harvested at the stated Days After Flowering (DAF) and the whole seeds were removed from the early pods under an Olympus SZ61 microscope (Melville, NY) and fresh-frozen in liquid nitrogen and stored at −80°C until extraction. More than 40 beans were harvested to represent the expression level of each line. For older stages, the seeds were shelled, pooled together, and then sorted by weight range. The seeds were first dissected to separate the seed coat and the cotyledon. The seed coats were frozen in liquid nitrogen for 10 minutes and stored in the freezer (−80°C) until they were lyophilized.

Total RNA was isolated by standard methods using phenol and chloroform extractions [28] and precipitated with ethanol but without lithium chloride in order to preserve small RNAs.

### Small RNA Sequencing and Data Analysis

Small RNA libraries and high throughput sequencing were performed with the Genome Analyzer-II and HiSeq-2000 (Illumina, San Diego, CA) by the Keck Center (University of Illinois, Urbana, IL) using standard Illumina protocols. Some of the sequences were barcoded for sequencing within a single lane. Generally, a total of ten to eighty million reads were obtained from these deep sequencing libraries. Adapter trimming was performed using Illumina’s Flicker pipeline which finds the presence of the adapter in each read by finding the best alignment of the adapter to the read, and removing it from the read. The sizes of the small RNAs after adapter trimming ranged from 14 to 33 nucleotides with the majority in the range of 18 to 25 nucleotides. Adapter trimmed sequences were compared to obtain the number and occurrences of unique sequences. Alignments of siRNA sequences to *CHS* Glyma models (Phytozome, Joint Genome Institute) from the Williams 82 reference genome of *Glycine max* [27] and from sequenced BACs including BAC77G7a, accession EF623858, containing the *I* locus clusters [7], [8] were performed using the Bowtie program [29]. Alignments were made to individual *CHS* sequences with no mismatches allowed. Small RNA sequencing data was normalized in reads per million (RPM).

### Accession Numbers

The data have been entered into Gene Expression Omnibus under series GSE43348 for the 18 samples of the developmental series small RNA shown in Table 1 and GSE49708 for the small RNA sequences from the various tissue samples shown in Table 2.

## Supporting Information

Figure S1 **The Total Counts of** ***CHS*** **siRNAs for Each** ***CHS*** **Gene Reveal Biogenesis of** ***CHS7/8*** **siRNAs is Dramatically Increased during Seed Coat Development in Biological Repeats of Eight Stages.** *CHS* siRNAs from small RNA libraries of ten developmental stages of the cultivar Williams *i^i^* were filtered to identify those with 100% identity to individual *CHS* genes as indicated by the color chart. Numbers above the bar indicate the total counts of *CHS4* (red) and *CHS7* (blue) siRNAs. Developmental stages are whole seed from (a) 4 DAF (Days After Flowering); (b) 12–14 DAF; and (c) 22–24 DAF; seed coats dissected from immature green seed of fresh weight (d) 5–6 mg; (e) 10–25 mg, no repeat data; (f) 25–50 mg, no repeat data; (g) 50–75 mg; (h) 75–100 mg; (i) 200–300 mg; and seed coats from (j) 300–400 mg yellow, desiccating seed.
(PDF)

Figure S2 **Size Distributions of** ***CHS*** **siRNAs in Biological Repeats of Eight Stages of Seed Coat Development.** *CHS* siRNAs from small RNA libraries of the same eight developmental stages as described in Supplemental Figure 1 were analyzed for their size distributions between 18 and 25 nt. The normalized total counts in reads per million (RPM) are shown for each *CHS* gene according to the color chart.
(PDF)

Figure S3 **Normalized Total Counts of** ***CHS*** **siRNAs that Match Either** ***CHS4*** **or** ***CHS7*** **with 100% Identity in Biological Repeats of Eight Stages of Seed Coat Development.** The 21-nt secondary *CHS7* siRNAs are amplified to high levels relative to the *CHS4* 21-nt or 22-nt siRNAs. Developmental stages are indicated.
(PDF)

Figure S4 **Alignment of** ***CHS4*** **and** ***CHS7*** **Coding Regions Shows that Polymorphisms are Widely Dispersed throughout the Two Sequences.** The longest contiguous sequences, which align to both genes without mismatch, are only 20 nt. These characteristics of the *CHS* gene family enable us to distinguish primary *CHS4* and secondary *CHS7* siRNAs by filtering their sequences to obtain only those with 100% identity. Primary *CHS4* siRNAs with one- or two-base mismatches to the target *CHS7* transcripts enable down-regulation of the target sequence.
(PDF)

Table S1 **Very Few** ***CHS*** **siRNAs Map with 100% Identity to Both Sub-groups of** ***CHS1/2/3/4/5/6/9*** **(Group 1) and** ***CHS7/8*** **(Group 2) Genes.** *CHS* siRNAs from small RNA libraries of the same ten developmental stages A–J as described in Figure 1 were analyzed for their size distributions between 18 and 25 nt. The normalized total counts in reads per million (RPM) are shown for each *CHS* gene. The *CHS* siRNAs that matched to a *CHS* gene within Group 1 (containing *CHS1/2/3/4/5/6/9*) were mapped to Group 2 targets containing *CHS7* and *CHS8* using Bowtie alignments and allowing no mismatches (unshaded line) or up to two mismatches (shaded line). Likewise, Group 2 *CHS* siRNAs that matched *CHS7* and *CHS8* were aligned to Group 1 targets (*CHS1/2/3/4/5/6/9*) allowing either zero or up to two mismatches. These data show that very few *CHS* siRNA were due to multi-matching siRNAs between the two groups when no mismatches are allowed but that significant numbers were multi-matching if up to 2 mismatches were allowed. Thus, imposing 100% identity enables the opportunity to distinguish *CHS1/3/4* primary siRNAs originating from the *CHS1-3-4* clusters at the *I* locus from the target *CHS7/8* secondary siRNAs.
(PDF)
