# Supplementary figures and images for: The Transition from Primary siRNAs to Amplified Secondary siRNAs That Regulate Chalcone Synthase During Development of Glycine max Seed Coats
Source: PLoS One. 2013 Oct 21;8(10):e76954. doi: 10.1371/journal.pone.0076954 (PMC3804491; doi:10.1371/journal.pone.0076954)

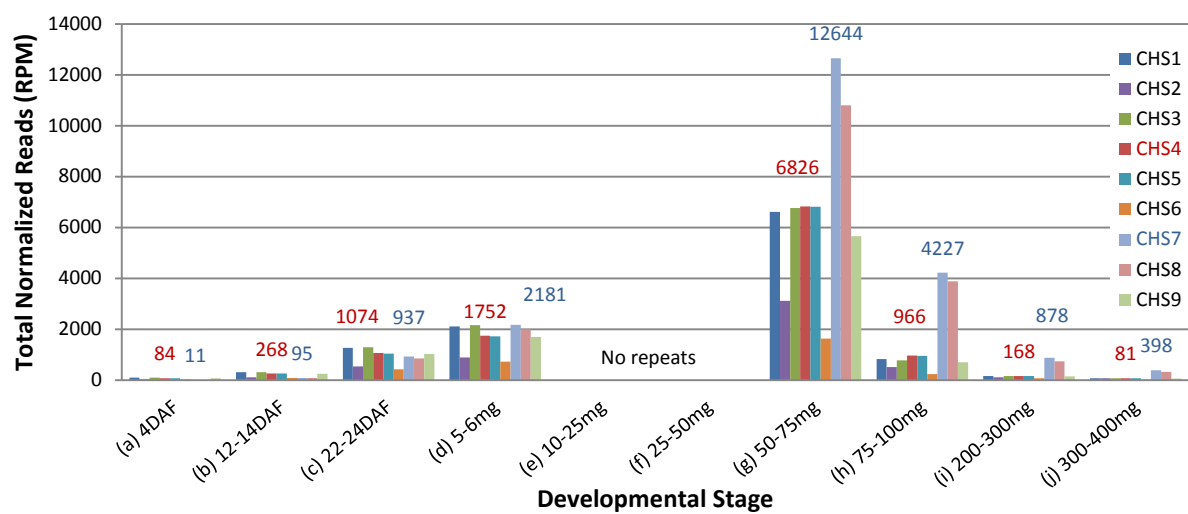

Supplement: Figure S1 — The Total Counts of CHS siRNAs for Each CHS Gene Reveal Biogenesis of CHS7/8 siRNAs is Dramatically Increased during Seed Coat Development in Biological Repeats of Eight Stages. CHS siRNAs from small RNA libraries of ten developmental stages of the cultivar Williams ii were filtered to identify those with 100% identity to individual CHS genes as indicated by the color chart. Numbers above the bar indicate the total counts of CHS4 (red) and CHS7 (blue) siRNAs. Developmental stages are whole seed from (a) 4 DAF (Days After Flowering); (b) 12–14 DAF; and (c) 22–24 DAF; seed coats dissected from immature green seed of fresh weight (d) 5–6 mg; (e) 10–25 mg, no repeat data; (f) 25–50 mg, no repeat data; (g) 50–75 mg; (h) 75–100 mg; (i) 200–300 mg; and seed coats from (j) 300–400 mg yellow, desiccating seed. (PDF) [file pone.0076954.s001.pdf]

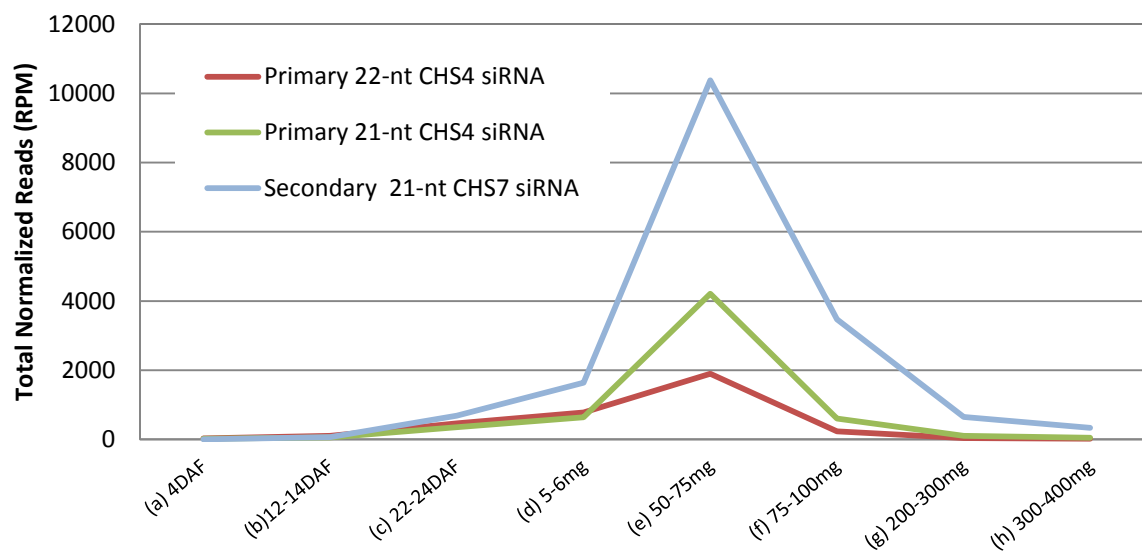

Supplement: Figure S3 — Normalized Total Counts of CHS siRNAs that Match Either CHS4 or CHS7 with 100% Identity in Biological Repeats of Eight Stages of Seed Coat Development. The 21-nt secondary CHS7 siRNAs are amplified to high levels relative to the CHS4 21-nt or 22-nt siRNAs. Developmental stages are indicated. (PDF) [file pone.0076954.s003.pdf]
