# Supplementary material for: The Transition from Primary siRNAs to Amplified Secondary siRNAs That Regulate Chalcone Synthase During Development of Glycine max Seed Coats
Source: PLoS One. 2013 Oct 21;8(10):e76954. doi: 10.1371/journal.pone.0076954 (PMC3804491; doi:10.1371/journal.pone.0076954)
Supplement: Figure S2 — Size Distributions of CHS siRNAs in Biological Repeats of Eight Stages of Seed Coat Development. CHS siRNAs from small RNA libraries of the same eight developmental stages as described in Supplemental Figure 1 were analyzed for their size distributions between 18 and 25 nt. The normalized total counts in reads per million (RPM) are shown for each CHS gene according to the color chart. (PDF) [file pone.0076954.s002.pdf]

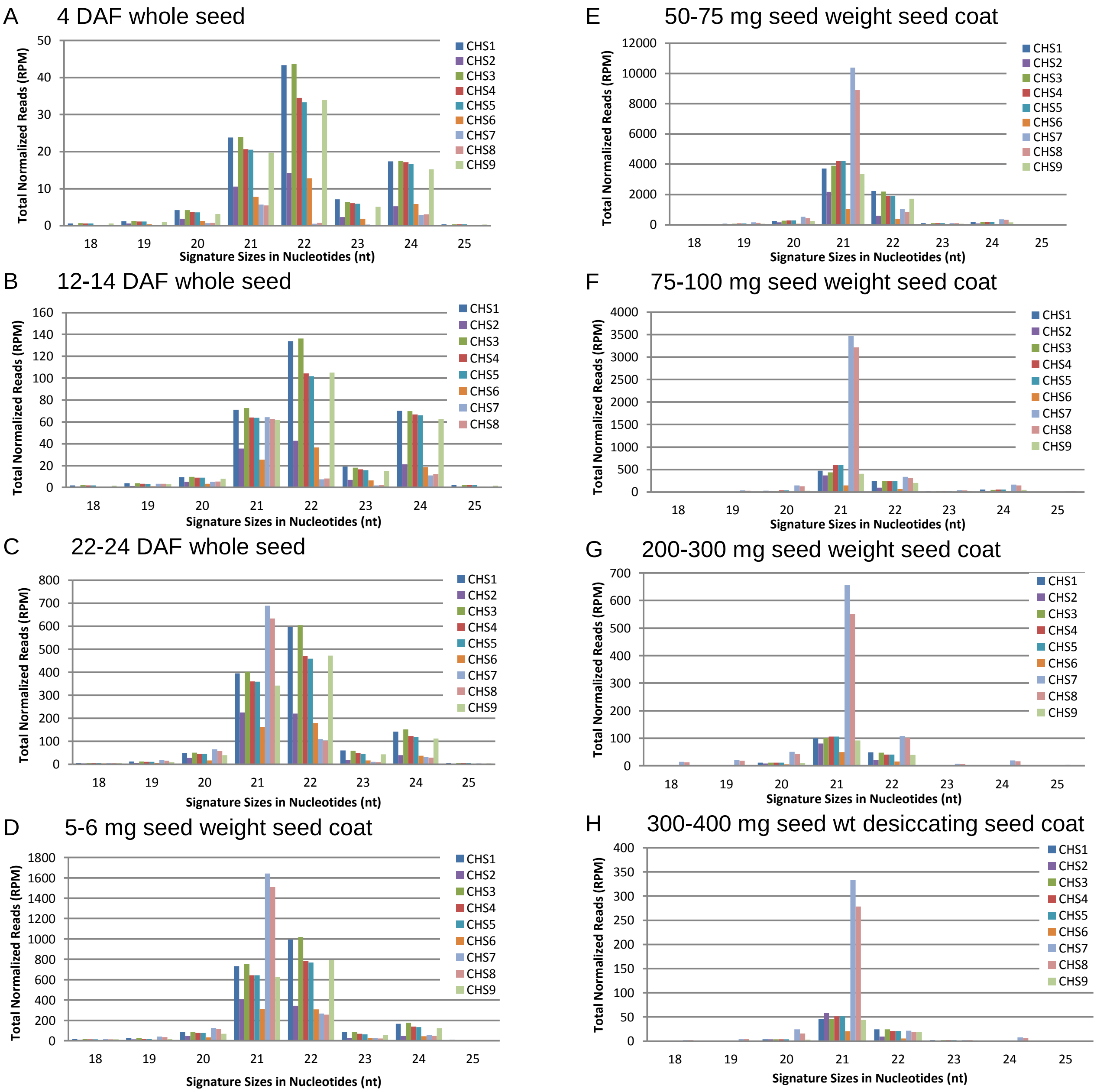
A
4 DAF whole seed
Total Normalized Reads (RPM)
0
10
20
30
40
50
18
19
20
21
22
23
24
25
Signature Sizes in Nucleotides (nt)
CHS1
CHS2
CHS3
CHS4
CHS5
CHS6
CHS7
CHS8
CHS9
B
12-14 DAF whole seed
Total Normalized Reads (RPM)
0
20
40
60
80
100
120
140
160
18
19
20
21
22
23
24
25
Signature Sizes in Nucleotides (nt)
CHS1
CHS2
CHS3
CHS4
CHS5
CHS6
CHS7
CHS8
C
22-24 DAF whole seed
Total Normalized Reads (RPM)
0
100
200
300
400
500
600
700
800
18
19
20
21
22
23
24
25
Signature Sizes in Nucleotides (nt)
CHS1
CHS2
CHS3
CHS4
CHS5
CHS6
CHS7
CHS8
CHS9
D
5-6 mg seed weight seed coat
Total Normalized Reads (RPM)
0
200
400
600
800
1000
1200
1400
1600
1800
18
19
20
21
22
23
24
25
Signature Sizes in Nucleotides (nt)
CHS1
CHS2
CHS3
CHS4
CHS5
CHS6
CHS7
CHS8
CHS9
E
50-75 mg seed weight seed coat
Total Normalized Reads (RPM)
0
2000
4000
6000
8000
10000
12000
18
19
20
21
22
23
24
25
Signature Sizes in Nucleotides (nt)
CHS1
CHS2
CHS3
CHS4
CHS5
CHS6
CHS7
CHS8
CHS9
F
75-100 mg seed weight seed coat
Total Normalized Reads (RPM)
0
500
1000
1500
2000
2500
3000
3500
4000
18
19
20
21
22
23
24
25
Signature Sizes in Nucleotides (nt)
CHS1
CHS2
CHS3
CHS4
CHS5
CHS6
CHS7
CHS8
CHS9
G
200-300 mg seed weight seed coat
Total Normalized Reads (RPM)
0
100
200
300
400
500
600
700
18
19
20
21
22
23
24
25
Signature Sizes in Nucleotides (nt)
CHS1
CHS2
CHS3
CHS4
CHS5
CHS6
CHS7
CHS8
CHS9
H
300-400 mg seed wt desiccating seed coat
Total Normalized Reads (RPM)
0
50
100
150
200
250
300
350
400
18
19
20
21
22
23
24
25
Signature Sizes in Nucleotides (nt)
CHS1
CHS2
CHS3
CHS4
CHS5
CHS6
CHS7
CHS8
CHS9
