# Supplementary material for: The Transition from Primary siRNAs to Amplified Secondary siRNAs That Regulate Chalcone Synthase During Development of Glycine max Seed Coats
Source: PLoS One. 2013 Oct 21;8(10):e76954. doi: 10.1371/journal.pone.0076954 (PMC3804491; doi:10.1371/journal.pone.0076954)
Supplement: Figure S4 — Alignment of CHS4 and CHS7 Coding Regions Shows that Polymorphisms are Widely Dispersed throughout the Two Sequences. The longest contiguous sequences, which align to both genes without mismatch, are only 20 nt. These characteristics of the CHS gene family enable us to distinguish primary CHS4 and secondary CHS7 siRNAs by filtering their sequences to obtain only those with 100% identity. Primary CHS4 siRNAs with one- or two-base mismatches to the target CHS7 transcripts enable down-regulation of the target sequence. (PDF) [file pone.0076954.s004.pdf]

```
Query  200   GATGGTGAGTGTTGAAGAGATTCGTAAGGCGCAACGTGCAGAAGGCCCTGCCACTGTCAT  259
             |||||| || || |  |||||  |  |||| ||| | ||||||||||| || ||  || |
Sbjct  200   GATGGTTAGCGTAGCTGAGATCAGGCAGGCACAAAGGGCAGAAGGCCCAGCAACCATCCT  259

Query  260   GGCTATTGGCACCGCCACTCCTCCCAACTGCGTGGATCAGAGTACCTATCCTGACTATTA  319
              || ||||| || || |  || || ||| | || |||||||| ||||||||||| || ||
Sbjct  260   TGCCATTGGAACTGCAAACCCACCAAACCGTGTTGATCAGAGCACCTATCCTGATTACTA  319

Query  320   TTTCCGCATCACCAACAGCGAGCACATGACCGAGCTCAAAGAAAAATTCAAACGCATGTG  379
              ||| | ||||||||||| || ||||||||||||||||||| |||||  | ||||||||
Sbjct  320   CTTCAGAATCACCAACAGTGACCACATGACCGAGCTCAAAGAGAAATTTCAGCGCATGTG  379

Query  380   TGATAAGTCGATGATTAAGAAGCGATACATGTACTTAAACGAAGAGATCCTGAAGGAGAA  439
             ||| ||||| ||||| |||| | |||| |||||| |||||||||||||| |||| |||||
Sbjct  380   TGACAAGTCTATGATCAAGACGAGATATATGTACCTAAACGAAGAGATCTTGAAAGAGAA  439

Query  440   TCCCAGTGTTTGTGCATATATGGCACCTTCGTTGGATGCAAGGCAAGACATGGTGGTTAT  499
             ||| |   | ||||| || ||||||||||| |||||||| |||||||||||||||||  |
Sbjct  440   TCCAAACATGTGTGCTTACATGGCACCTTCTTTGGATGCTAGGCAAGACATGGTGGTGGT  499

Query  500   GGAGGTACCAAAGTTGGGAAAAGAGGCTGCAACTAAGGCAATCAAGGAATGGGGTCAACC  559
              |||||||||||| | || ||||||||||||   ||||| || ||||| ||||| || ||
Sbjct  500   AGAGGTACCAAAGCTAGGGAAAGAGGCTGCAGTAAAGGCCATAAAGGAGTGGGGCCAGCC  559

Query  560   CAAGTCCAAGATTACCCATCTCATCTTTTGCACCACTAGTGGTGTCGACATGCCTGGTGC  619
              ||||| |||||||||||  | ||||| ||||||||||| ||||| ||||||||||||||
Sbjct  560   AAAGTCAAAGATTACCCACTTGATCTTCTGCACCACTAGCGGTGTGGACATGCCTGGTGC  619

Query  620   TGATTATCAGCTCACTAAACTATTAGGCCTTCGTCCCTCCGTCAAGCGTTACATGATGTA  679
             |||||| || ||||| |||| ||| |||||||| || |  || ||| | |||||||||||
Sbjct  620   TGATTACCAACTCACCAAACAATTGGGCCTTCGCCCTTATGTGAAGAGGTACATGATGTA  679

Query  680   CCAACAAGGCTGCTTTGCCGGTGGCACGGTGCTTCGTTTGGCCAAAGACCTCGCTGAAAA  739
             ||||||||| |||||||| ||||||||||| |||||||||||||| ||  | ||||| ||
Sbjct  680   CCAACAAGGTTGCTTTGCAGGTGGCACGGTTCTTCGTTTGGCCAAGGATTTGGCTGAGAA  739

Query  740   CAACAAGGGTGCTCGCGTGCTTGTCGTTTGTTCTGAGATCACCGCAGTCACATTCCGCGG  799
             |||||||||||| || |||||||| || || ||||||||||| |||||||||||||| ||
Sbjct  740   CAACAAGGGTGCACGTGTGCTTGTTGTCTGCTCTGAGATCACTGCAGTCACATTCCGTGG  799

Query  800   CCCAACTGACACCCATCTTGATAGCCTTGTGGGTCAAGCCTTGTTTGGAGATGGTGCAGC  859
              |||| |||||| || |||||||| |||||||| ||||| |||||||||||||| || ||
Sbjct  800   GCCAAGTGACACTCACCTTGATAGTCTTGTGGGCCAAGCATTGTTTGGAGATGGAGCTGC  859
```

```
Query  860   CGCTGTCATTGTTGGATCAGACCCCTT---ACCAGTTGAAAAGCCTTTGTTTCAGCTTGT  916
              || ||||||||||| || |||||  |   || |||||| |||||||||| | |||||||
Sbjct  860   TGCAGTCATTGTTGGTTCTGACCCAATTCCACAAGTTGAGAAGCCTTTGTATGAGCTTGT  919

Query  917   CTGGACTGCCCAGACAATCCTTCCAGACAGTGAAGGGGCTATTGATGGACACCTTCGCGA  976
              |||||||| || |||||   ||||||||||||||| |||||||||||||||||||| ||
Sbjct  920   TTGGACTGCACAAACAATTGCTCCAGACAGTGAAGGTGCTATTGATGGACACCTTCGTGA  979

Query  977   AGTTGGTCTCACTTTCCATCTCCTCAAGGATGTTCCTGGACTCATCTCCAAGAATATTGA  1036
             |||||| ||||| || || ||||||||||||||||| ||  |  |||| ||||| |||||
Sbjct  980   AGTTGGACTCACATTTCACCTCCTCAAGGATGTTCCCGGGATTGTCTCAAAGAACATTGA  1039

Query  1037  GAAGGCCTTGGTTGAAGCCTTCCAACCCTTGGGAATCTCCGATTACAATTCTATCTTCTG  1096
              |||||  |  |||| || ||| | || |||   ||||| |||||||| || ||||| ||
Sbjct  1040  TAAGGCACTTTTTGAGGCTTTCAACCCATTGAACATCTCTGATTACAACTCCATCTTTTG  1099

Query  1097  GATTGCACACCCTGGTGGACCCGCAATTTTGGACCAAGTGGAGGCTAAGTTAGGCTTGAA  1156
             |||||||||||||||||| || || ||||| |||||||| |||   ||||| ||  | ||
Sbjct  1100  GATTGCACACCCTGGTGGGCCTGCGATTTTAGACCAAGTTGAGCAAAAGTTGGGTCTCAA  1159

Query  1157  GCCTGAAAAAATGGAAGCTACTAGGCATGTGCTCAGCGAGTATGGTAACATGTCAAGTGC  1216
              ||||| || ||| | || |||||  ||||||| || || ||||| ||||||||||||||
Sbjct  1160  ACCTGAGAAGATGAAGGCCACTAGAGATGTGCTTAGTGAATATGGGAACATGTCAAGTGC  1219

Query  1217  ATGTGTGCTATTCATCTTGGATCAAATGCGGAAGAAATCAATAGAAAATGGACTTGGCAC  1276
              ||||| || |||||||||||| | ||| ||| ||||||    |||||||||| |   ||
Sbjct  1220  TTGTGTTCTTTTCATCTTGGATGAGATGAGGAGGAAATCTGCTGAAAATGGACATAAAAC  1279

Query  1277  AACCGGCGAAGGCCTTGACTGGGGTGTGCTATTTGGTTTCGGTCCTGGACTCACTGTTGA  1336
              || || ||||| ||||| ||||||||| | || ||||| || |||||||| ||  ||||
Sbjct  1280  CACAGGTGAAGGACTTGAATGGGGTGTGTTGTTCGGTTTTGGACCTGGACTTACCATTGA  1339

Query  1337  GACTGTTGT  1345
              ||||||||
Sbjct  1340  AACTGTTGT  1348
```

Figure S4. Alignment of *CHS4* and *CHS7* Coding Regions Shows that Polymorphisms are Widely Dispersed throughout the Two Sequences. The longest contiguous sequences, which align to both genes without mismatch, are only 20 nt. These characteristics of the *CHS* gene family enable us to distinguish primary *CHS4* and secondary *CHS7* siRNAs by filtering their sequences to obtain only those with 100% identity. Primary *CHS4* siRNAs with one- or two-base mismatches to the target *CHS7* transcripts enable down-regulation of the target sequence.
