# Supplementary material for: The Transition from Primary siRNAs to Amplified Secondary siRNAs That Regulate Chalcone Synthase During Development of Glycine max Seed Coats
Source: PLoS One. 2013 Oct 21;8(10):e76954. doi: 10.1371/journal.pone.0076954 (PMC3804491; doi:10.1371/journal.pone.0076954)
Supplement: Table S1 — Very Few CHS siRNAs Map with 100% Identity to Both Sub-groups of CHS1/2/3/4/5/6/9 (Group 1) and CHS7/8 (Group 2) Genes. CHS siRNAs from small RNA libraries of the same ten developmental stages A–J as described in Figure 1 were analyzed for their size distributions between 18 and 25 nt. The normalized total counts in reads per million (RPM) are shown for each CHS gene. The CHS siRNAs that matched to a CHS gene within Group 1 (containing CHS1/2/3/4/5/6/9) were mapped to Group 2 targets containing CHS7 and CHS8 using Bowtie alignments and allowing no mismatches (unshaded line) or up to two mismatches (shaded line). Likewise, Group 2 CHS siRNAs that matched CHS7 and CHS8 were aligned to Group 1 targets (CHS1/2/3/4/5/6/9) allowing either zero or up to two mismatches. These data show that very few CHS siRNA were due to multi-matching siRNAs between the two groups when no mismatches are allowed but that significant numbers were multi-matching if up to 2 mismatches were allowed. Thus, imposing 100% identity enables the opportunity to distinguish CHS1/3/4 primary siRNAs originating from the CHS1-3-4 clusters at the I locus from the target CHS7/8 secondary siRNAs. (PDF) [file pone.0076954.s005.pdf]

A. 4 DAF whole seed

Group		Group 1							Group 2	
**nt**	**Mismatch**	**CHS1**	**CHS2**	**CHS3**	**CHS4**	**CHS5**	**CHS6**	**CHS9**	**CHS7**	**CHS8**
**18**	0	0.00	0.00	0.00	0.00	0.00	0.00	0.00	0.00	0.00
	2	0.58	0.16	0.16	0.16	0.16	0.16	0.16	0.00	0.00
**19**	0	0.00	0.00	0.00	0.00	0.00	0.00	0.00	0.00	0.00
	2	1.07	0.00	0.08	0.08	0.08	0.00	0.08	0.00	0.00
**20**	0	0.08	0.08	0.08	0.08	0.08	0.08	0.08	0.08	0.00
	2	1.23	0.08	0.25	0.16	0.16	0.08	0.16	0.25	0.16
**21**	0	0.00	0.00	0.00	0.00	0.00	0.08	0.00	0.08	0.00
	2	29.76	1.15	3.70	3.78	3.78	0.74	2.63	2.22	2.14
**22**	0	0.00	0.00	0.00	0.00	0.00	0.00	0.00	0.00	0.00
	2	62.40	2.96	7.40	6.74	6.74	1.56	4.85	0.00	0.00
**23**	0	0.00	0.00	0.00	0.00	0.00	0.00	0.00	0.00	0.00
	2	2.30	0.08	0.25	0.25	0.25	0.08	0.25	0.00	0.08
**24**	0	0.00	0.00	0.00	0.00	0.00	0.00	0.00	0.00	0.00
	2	14.47	0.90	1.81	1.73	1.73	0.66	1.73	0.41	0.49
**25**	0	0.00	0.00	0.00	0.00	0.00	0.00	0.00	0.00	0.00
	2	0.00	0.08	0.08	0.08	0.08	0.08	0.08	0.00	0.08

B. 12-14 DAF whole seed

Group		Group 1							Group 2	
**nt**	**Mismatch**	**CHS1**	**CHS2**	**CHS3**	**CHS4**	**CHS5**	**CHS6**	**CHS9**	**CHS7**	**CHS8**
**18**	0	0.00	0.00	0.00	0.00	0.00	0.46	0.00	0.00	0.00
	2	0.53	0.38	0.53	0.53	0.53	0.61	0.53	0.84	0.61
**19**	0	0.00	0.00	0.00	0.00	0.00	0.00	0.00	0.00	0.00
	2	0.99	0.30	0.99	0.99	0.99	0.00	0.84	0.61	0.61
**20**	0	0.30	0.00	0.30	0.30	0.30	0.00	0.00	0.00	0.30
	2	1.14	0.38	1.14	1.14	1.14	0.84	0.84	0.76	1.37
**21**	0	2.13	0.00	0.76	0.76	0.76	4.34	0.00	4.34	0.76
	2	27.56	17.21	28.10	28.10	28.10	11.88	24.21	52.23	49.87
**22**	0	0.00	0.00	1.37	1.37	1.37	0.23	0.00	0.23	1.37
	2	57.79	24.97	58.78	50.48	50.48	13.78	43.02	2.89	3.96
**23**	0	0.00	0.00	0.00	0.00	0.00	0.00	0.00	0.00	0.00
	2	2.13	0.76	2.74	1.68	1.68	0.76	1.68	0.00	0.00
**24**	0	0.00	0.00	0.00	0.00	0.00	0.00	0.00	0.00	0.00
	2	13.40	4.26	11.95	10.58	10.58	2.51	9.97	1.98	1.68
**25**	0	0.00	0.00	0.00	0.00	0.00	0.00	0.00	0.00	0.00
	2	0.00	0.00	0.00	0.00	0.00	0.00	0.00	0.00	0.00

C. 22-24 DAF whole seed

Group		Group 1							Group 2	
**nt**	**Mismatch**	**CHS1**	**CHS2**	**CHS3**	**CHS4**	**CHS5**	**CHS6**	**CHS9**	**CHS7**	**CHS8**
**18**	0	0.00	0.00	0.00	0.00	0.00	0.00	0.00	0.00	0.00
	2	0.00	0.00	0.00	0.00	0.00	0.00	0.00	0.10	0.19
**19**	0	0.00	0.00	0.00	0.00	0.00	0.00	0.00	0.00	0.00
	2	0.10	0.00	0.10	0.10	0.10	0.00	0.10	0.19	0.19
**20**	0	0.00	0.00	0.00	0.00	0.00	0.10	0.00	0.10	0.00
	2	0.29	0.29	0.19	0.19	0.19	0.19	0.10	0.29	0.19
**21**	0	0.00	0.00	0.00	0.00	0.00	0.00	0.00	0.00	0.00
	2	2.71	1.94	2.52	2.71	2.71	1.06	2.13	14.23	13.74
**22**	0	0.19	0.00	0.19	0.19	0.19	0.00	0.00	0.00	0.19
	2	9.48	5.03	9.39	6.77	6.77	3.19	6.19	0.58	0.58
**23**	0	0.00	0.00	0.00	0.00	0.00	0.00	0.00	0.00	0.00
	2	0.19	0.29	0.19	0.10	0.10	0.19	0.19	0.10	0.00
**24**	0	0.00	0.00	0.00	0.00	0.00	0.00	0.00	0.00	0.00
	2	4.55	2.61	4.74	4.06	4.06	1.84	4.06	0.87	0.77
**25**	0	0.00	0.00	0.00	0.00	0.00	0.00	0.00	0.00	0.00
	2	0.29	0.10	0.19	0.19	0.19	0.00	0.19	0.00	0.00

D. 5-6mg seed weight seed coat

Group		Group 1							Group 2	
**nt**	**Mismatch**	**CHS1**	**CHS2**	**CHS3**	**CHS4**	**CHS5**	**CHS6**	**CHS9**	**CHS7**	**CHS8**
**18**	0	0.11	0.22	0.11	0.11	0.11	0.56	0.11	0.62	0.22
	2	1.23	0.95	1.57	1.62	1.62	0.73	1.62	4.09	2.86
**19**	0	0.17	0.28	0.17	0.17	0.17	1.06	0.06	1.23	0.45
	2	9.97	3.02	4.98	4.42	4.42	2.69	3.86	10.75	8.45
**20**	0	0.39	0.56	0.39	0.39	0.39	10.19	0.11	10.58	0.90
	2	22.45	13.66	22.79	20.94	20.94	15.62	17.13	57.05	42.89
**21**	0	0.17	0.17	0.17	0.17	0.17	22.40	0.11	22.56	0.45
	2	71.72	41.15	72.39	69.65	69.65	41.49	61.53	196.13	156.88
**22**	0	0.73	0.17	0.73	0.73	0.73	2.13	0.00	2.30	0.90
	2	83.31	37.01	82.75	73.35	73.35	22.68	62.43	19.04	15.96
**23**	0	0.00	0.00	0.00	0.00	0.00	0.34	0.00	0.34	0.00
	2	7.45	2.91	7.84	6.55	6.55	2.24	5.26	2.97	1.90
**24**	0	0.00	0.00	0.00	0.00	0.00	0.73	0.00	0.73	0.00
	2	11.20	4.31	11.48	9.97	9.97	2.97	8.29	4.09	2.80
**25**	0	0.00	0.00	0.00	0.00	0.00	0.11	0.00	0.11	0.00
	2	0.67	0.17	0.73	0.62	0.62	0.34	0.50	0.67	0.62

E. 10-25mg seed weight seed coat

Group		Group 1							Group 2	
**nt**	**Mismatch**	**CHS1**	**CHS2**	**CHS3**	**CHS4**	**CHS5**	**CHS6**	**CHS9**	**CHS7**	**CHS8**
**18**	0	0.08	0.08	0.08	0.08	0.08	0.41	0.00	0.41	0.17
	2	1.65	0.99	1.90	1.82	1.82	0.91	1.65	5.71	4.47
**19**	0	0.25	0.25	0.25	0.25	0.25	0.91	0.17	0.91	0.25
	2	5.46	2.65	5.79	5.30	5.30	2.15	4.80	15.14	12.91
**20**	0	0.25	0.66	0.25	0.25	0.25	8.94	0.08	9.27	0.99
	2	19.69	14.07	19.86	18.62	18.62	14.89	16.71	67.60	57.18
**21**	0	0.99	1.99	0.99	0.99	0.99	67.44	1.32	69.42	4.30
	2	257.67	180.47	255.77	260.73	260.73	146.54	239.71	1186.33	1019.76
**22**	0	2.81	0.50	2.81	2.81	2.81	6.37	0.00	6.87	3.31
	2	251.63	106.58	248.82	228.46	228.46	60.40	193.54	97.39	87.21
**23**	0	0.00	0.00	0.00	0.00	0.00	0.50	0.00	0.50	0.00
	2	12.00	4.55	12.08	10.34	10.34	3.06	8.19	8.11	6.70
**24**	0	0.00	0.00	0.00	0.00	0.00	4.55	0.00	4.55	0.00
	2	10.76	11.34	29.95	28.38	28.38	10.10	22.92	29.29	19.61
**25**	0	0.00	0.00	0.00	0.00	0.00	0.58	0.00	0.58	0.00
	2	1.16	0.58	0.99	0.91	0.91	0.74	0.83	3.48	1.74

F. 25-50mg seed weight seed coat

Group		Group 1							Group 2	
**nt**	**Mismatch**	**CHS1**	**CHS2**	**CHS3**	**CHS4**	**CHS5**	**CHS6**	**CHS9**	**CHS7**	**CHS8**
**18**	0	0.08	0.25	0.08	0.08	0.08	0.25	0.00	0.74	0.66
	2	2.65	1.90	2.81	2.48	2.48	1.41	2.23	8.27	7.28
**19**	0	0.17	0.08	0.17	0.17	0.17	0.83	0.08	0.91	0.17
	2	4.55	2.73	4.96	4.63	4.63	1.57	4.22	18.54	16.63
**20**	0	0.83	1.08	0.83	0.83	0.83	12.41	0.50	12.66	1.57
	2	17.87	13.82	17.71	16.05	16.05	17.46	14.32	83.08	66.44
**21**	0	1.16	2.98	1.16	1.16	1.16	69.65	0.58	72.63	4.71
	2	242.11	173.77	237.07	247.66	247.66	141.91	231.77	1449.21	1265.18
**22**	0	0.50	0.33	0.50	0.50	0.50	8.44	0.00	8.77	0.83
	2	142.90	67.93	145.38	135.04	135.04	41.95	117.33	119.15	106.58
**23**	0	0.00	0.08	0.00	0.00	0.00	0.50	0.00	0.58	0.08
	2	7.20	2.81	7.28	6.87	6.87	1.90	5.13	12.16	10.67
**24**	0	0.00	0.00	0.00	0.00	0.00	0.91	0.00	3.23	0.00
	2	21.93	7.12	21.84	20.02	20.02	6.54	15.64	31.36	22.67
**25**	0	0.00	0.00	0.00	0.00	0.00	0.25	0.00	0.25	0.00
	2	0.83	0.58	0.91	0.99	0.99	0.74	0.99	3.23	1.90

G. 50-75mg seed weight seed coat

Group		Group 1							Group 2	
**nt**	**Mismatch**	**CHS1**	**CHS2**	**CHS3**	**CHS4**	**CHS5**	**CHS6**	**CHS9**	**CHS7**	**CHS8**
**18**	0	0.09	0.09	0.09	0.09	0.09	0.61	0.09	0.61	0.00
	2	4.52	2.96	5.04	4.95	4.95	2.09	4.35	20.25	16.51
**19**	0	0.17	0.35	0.17	0.17	0.17	1.74	0.09	2.00	0.52
	2	8.17	4.61	8.17	8.17	8.17	3.39	7.82	37.55	33.46
**20**	0	0.43	0.96	0.43	0.43	0.43	14.78	0.09	15.38	1.48
	2	26.77	20.95	26.95	25.73	25.73	24.86	22.95	138.20	118.47
**21**	0	1.74	3.04	1.74	1.74	1.74	75.53	1.74	78.58	6.52
	2	330.39	243.81	326.39	341.95	341.95	177.49	311.78	2083.93	1895.74
**22**	0	0.78	0.61	0.78	0.78	0.78	8.26	0.09	9.91	1.48
	2	197.57	97.70	200.44	185.05	185.05	55.28	157.67	188.53	170.54
**23**	0	0.00	0.09	0.00	0.00	0.00	0.96	0.00	1.04	0.09
	2	12.00	4.43	12.95	11.13	11.13	2.87	8.61	20.08	18.77
**24**	0	0.00	0.00	0.00	0.00	0.00	3.56	0.00	3.56	0.00
	2	33.55	11.13	33.81	30.68	30.68	9.13	25.47	39.38	30.34
**25**	0	0.00	0.00	0.00	0.00	0.00	0.61	0.00	0.61	0.00
	2	1.65	0.61	1.56	1.30	1.30	0.96	1.30	6.69	5.48

H. 75-100mg seed weight seed coat

Group		Group 1							Group 2	
**nt**	**Mismatch**	**CHS1**	**CHS2**	**CHS3**	**CHS4**	**CHS5**	**CHS6**	**CHS9**	**CHS7**	**CHS8**
**18**	0	0.00	0.00	0.00	0.00	0.00	0.29	0.00	0.29	0.00
	2	1.71	1.33	1.81	1.71	1.71	1.14	1.71	8.28	7.61
**19**	0	0.19	0.29	0.19	0.19	0.19	0.67	0.19	0.76	0.29
	2	3.33	2.00	3.24	2.66	2.66	1.90	2.76	14.28	12.28
**20**	0	0.10	0.48	0.10	0.10	0.10	4.28	0.00	4.66	0.57
	2	8.95	8.47	8.85	9.23	9.23	8.09	8.28	53.87	48.16
**21**	0	0.19	0.67	0.19	0.19	0.19	13.51	0.29	14.18	1.14
	2	44.54	37.31	43.87	46.06	46.06	29.98	42.26	460.44	436.45
**22**	0	0.00	0.38	0.00	0.00	0.00	1.52	0.00	1.90	0.38
	2	22.94	13.23	23.41	21.41	21.41	9.33	19.51	31.79	29.12
**23**	0	0.00	0.00	0.00	0.00	0.00	0.29	0.00	0.29	0.00
	2	1.24	0.38	1.52	1.43	1.43	0.57	1.14	6.47	6.00
**24**	0	0.00	0.00	0.00	0.00	0.00	0.38	0.00	0.38	0.00
	2	4.76	2.09	5.04	5.33	5.33	1.43	4.76	10.18	8.18
**25**	0	0.00	0.00	0.00	0.00	0.00	0.19	0.00	0.19	0.00
	2	0.57	0.38	0.86	0.38	0.38	0.48	0.38	2.00	1.33

I. 200-300mg seed weight seed coat

Group		Group 1							Group 2	
**nt**	**Mismatch**	**CHS1**	**CHS2**	**CHS3**	**CHS4**	**CHS5**	**CHS6**	**CHS9**	**CHS7**	**CHS8**
**18**	0	0.07	0.00	0.07	0.07	0.07	0.13	0.00	0.13	0.07
	2	0.20	0.07	0.20	0.20	0.20	0.13	0.13	2.15	1.75
**19**	0	0.07	0.00	0.07	0.07	0.07	0.34	0.00	0.40	0.13
	2	0.13	0.07	0.13	0.20	0.20	0.40	0.13	3.64	3.03
**20**	0	0.00	0.00	0.00	0.00	0.00	0.94	0.00	0.94	0.00
	2	0.88	0.61	0.74	0.61	0.61	1.08	0.54	8.15	6.13
**21**	0	0.00	0.47	0.00	0.00	0.00	5.52	0.00	5.99	0.47
	2	6.46	7.88	6.46	7.20	7.20	7.47	7.07	151.33	129.99
**22**	0	0.07	0.00	0.07	0.07	0.07	0.20	0.00	0.20	0.07
	2	4.38	1.62	4.51	4.11	4.11	1.08	2.83	16.29	15.15
**23**	0	0.00	0.00	0.00	0.00	0.00	0.00	0.00	0.00	0.00
	2	0.00	0.00	0.00	0.00	0.00	0.00	0.00	2.15	1.48
**24**	0	0.00	0.00	0.00	0.00	0.00	0.00	0.00	0.00	0.00
	2	1.01	0.13	0.94	0.94	0.94	0.07	0.88	4.04	2.49
**25**	0	0.00	0.00	0.00	0.00	0.00	0.00	0.00	0.00	0.00
	2	0.13	0.13	0.13	0.13	0.13	0.07	0.13	0.47	0.40

J. 300-400mg seed wt desiccating seed coat

Group		Group 1							Group 2	
**nt**	**Mismatch**	**CHS1**	**CHS2**	**CHS3**	**CHS4**	**CHS5**	**CHS6**	**CHS9**	**CHS7**	**CHS8**
**18**	0	0.00	0.00	0.00	0.00	0.00	0.12	0.00	0.12	0.12
	2	0.12	0.29	0.12	0.12	0.12	0.23	0.12	1.28	1.05
**19**	0	0.00	0.00	0.00	0.00	0.00	0.06	0.00	0.06	0.00
	2	0.17	0.23	0.17	0.17	0.17	0.12	0.06	1.86	1.51
**20**	0	0.00	0.06	0.00	0.00	0.00	0.23	0.00	0.23	0.12
	2	0.99	1.46	0.82	0.99	0.99	0.47	0.93	4.95	4.48
**21**	0	0.00	0.23	0.00	0.00	0.00	4.37	0.00	4.60	0.23
	2	9.90	16.42	9.20	9.14	9.14	7.51	8.50	126.05	107.30
**22**	0	0.06	0.06	0.06	0.06	0.06	0.17	0.00	0.23	0.12
	2	4.54	3.38	4.72	4.08	4.08	1.40	2.91	11.82	11.35
**23**	0	0.00	0.00	0.00	0.00	0.00	0.00	0.00	0.00	0.00
	2	0.41	0.06	0.29	0.41	0.41	0.00	0.23	0.93	0.41
**24**	0	0.00	0.00	0.00	0.00	0.00	0.06	0.00	0.06	0.00
	2	0.41	0.29	0.41	0.35	0.35	0.17	0.29	1.22	0.52
**25**	0	0.00	0.00	0.00	0.00	0.00	0.06	0.00	0.06	0.00
	2	0.35	0.06	0.17	0.06	0.06	0.06	0.06	0.52	0.29

Table S1. Very Few *CHS* siRNAs Map with 100% Identity to Both Sub-groups of *CHS1/2/3/4/5/6/9* (Group 1) and *CHS7/8* (Group 2) Genes. *CHS* siRNAs from small RNA libraries of the same ten developmental stages A-J as described in Figure 1 were analyzed for their size distributions between 18 and 25 nt. The normalized total counts in reads per million (RPM) are shown for each *CHS* gene. The *CHS* siRNAs that matched to a *CHS* gene within Group 1 (containing *CHS1/2/3/4/5/6/9*) were mapped to Group 2 targets containing *CHS7* and *CHS8* using Bowtie alignments and allowing no mismatches (unshaded line) or up to two mismatches (shaded line). Likewise, Group 2 *CHS* siRNAs that matched *CHS7* and *CHS8* were aligned to Group 1 targets (*CHS1/2/3/4/5/6/9*) allowing either zero or up to two mismatches. These data show that very few *CHS* siRNA were due to multi-matching siRNAs between the two groups when no mismatches are allowed but that significant numbers were multi-matching if up to 2 mismatches were allowed. Thus, imposing 100% identity enables the opportunity to distinguish *CHS1/3/4* primary siRNAs originating from the *CHS1-3-4* clusters at the *I* locus from the target *CHS7/8* secondary siRNAs.
